# Self-assembled *c*-oriented Ni(OH)_2_ films for enhanced electrocatalytic activity towards the urea oxidation reaction†

**DOI:** 10.1039/d3ra05538h

**Published:** 2023-10-10

**Authors:** Xinwei Dong, Chen Peng, Xu Zhao, Tao Zhang, Yansheng Liu, Guoxiao Xu, Jin Zhou, Fei Guo, Zhiqiang Yu, Xiaobo Jia

**Affiliations:** a School of Electronic Engineering, Liuzhou Key Laboratory of New Energy Vehicle Power Lithium Battery, Guangxi University of Science and Technology Liuzhou 545006 Guangxi China 100002409@gxust.edu.cn; b School of Computer Science and Technology, Guangxi University of Science and Technology Liuzhou 545006 Guangxi China

## Abstract

This study investigates the electrocatalytic properties of the transparent *c*-oriented Ni(OH)_2_ films self-assembled from colloidal 2D Ni(OH)_2_ nanosheets for urea oxidation. The synthesis process yields highly uniform close-packed superlattices with a dominant *c*-axis orientation. The self-assembled *c*-oriented Ni(OH)_2_ films exhibit advantageous electrocatalytic performance in urea oxidation, presenting significantly lower overpotentials and higher current densities compared to randomly distributed Ni(OH)_2_ particles. In-depth *in situ* impedance analysis and Raman spectroscopy demonstrate that the *c*-oriented Ni(OH)_2_ films possess a higher propensity for a Ni valence transition from +2 to +3 during the urea oxidation process. This finding provides crucial insights into the catalytic behavior and electronic transformations of *c*-oriented Ni(OH)_2_ films, shedding light on their superior electrocatalytic activity for urea oxidation. Overall, this study advances our understanding of urea electrooxidation mechanisms and contributes to the design of efficient urea electrocatalysts.

## Abstract 2

The self-assembled *c*-oriented Ni(OH)_2_ films exhibit exceptional electrocatalytic performance for urea oxidation compared to randomly distributed Ni(OH)_2_ particles.

## Graphical abstract

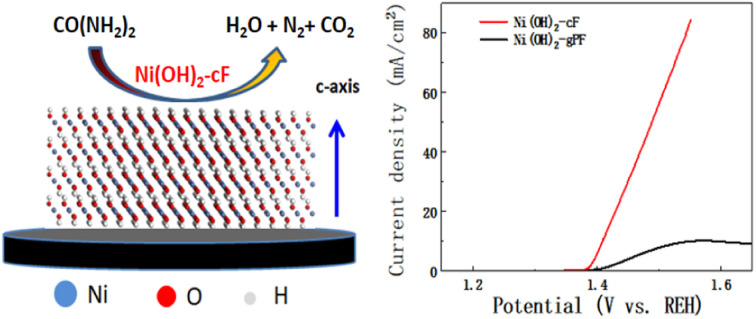

## Introduction

Urea, a highly abundant compound present in bio-waste and extensively utilized in the chemical industry, has been recognized as a potent renewable energy source. The central process in urea electrolysis, which encompasses hydrogen evolution, direct urea fuel cells, bio-waste remediation, and medical urea detection, is the electrochemical urea oxidation reaction (UOR). However, this process faces significant challenges due to its sluggish reaction kinetics, characterized by a complex six-electron and four-proton mechanism.^1–5^ Hence, the development of efficient electrocatalysts capable of reducing overpotentials, augmenting current densities, and ensuring long-term stability is of paramount importance.^6^

Nickel hydroxide (Ni(OH)_2_) is a standout as a potential catalyst for UOR owing to its advantageous catalytic characteristics, cost-effectiveness, and ubiquity.^7–12^ Despite extensive research on nickel-based catalysts, uncertainties surrounding catalytic activity, stability, and mechanistic understanding endure. In this regard, a variety of Ni(OH)_2_-based catalysts, such as atomically thick Ni(OH)_2_ nanosheets,^7^ Ni(OH)_2_ nanoflakes,^13^ variants of Ni(OH)_2_ material with diverse heteroatom dopants, exemplified by instances of Co^14,15^ and Fe^16^ dopants, and Ni(OH)_2_–carbon composites^17,18^ have been investigated. However, the practical application of Ni(OH)_2_ as an effective electrocatalyst for urea oxidation is thwarted by its restricted electrical conductivity and instability under severe electrochemical conditions.^18–22^ To circumvent these limitations, novel approaches are being explored to enhance the electrocatalytic efficiency of Ni(OH)_2_.

One promising approach involves self-assembly of colloidal two-dimensional (2D) Ni(OH)_2_ nanosheets into highly ordered films.^23–26^ The self-assembly patterns of colloidal nanocrystals are increasingly recognized as a valuable methodology for investigating the fundamental characteristics of highly uniform nanoparticles. In preceding work,^27^ we successfully engineered *c*-oriented NiFe-LDH films and Ni(OH)_2_ films using a colloid self-assembly method. The resultant films demonstrated superior electrocatalytic properties in the oxygen evolution reaction (OER) and ethanol electrooxidation reaction due to their orderly *c*-oriented superlattices, which led to elevated proton diffusion coefficients and significantly enhanced electrocatalytic activities.^27^ The findings from this research emphasize the conceivable potential of these self-assembled *c*-oriented films from colloidal nano particles for diverse applications in the field of electrocatalysis.^28,29^

This current investigation aims to thoroughly examine the UOR performance of transparent *c*-oriented Ni(OH)_2_ films self-assembled from colloidal 2D Ni(OH)_2_ nanosheets. The primary objective of this paper is to assess the potential of these films to enhance the electrocatalytic activity for the UOR and to investigate the underlying mechanisms that contribute to their superior electrocatalytic performance. In this study, our comprehensive analysis revealed that the *c*-oriented Ni(OH)_2_ films (Ni(OH)_2_-cF) showcased exceptional electrocatalytic efficiency. Ni(OH)_2_-cF, when applied to the glassy carbon electrode, demonstrated outstanding UOR electrocatalytic activity, resulting in a significant lower value of 180 mV in potential at a current density of 10 mA cm^−2^. This surpasses randomly distributed Ni(OH)_2_ films (Ni(OH)_2_-gPF) by a notable margin of 118 mV. Furthermore, our research has provided valuable insights into the governing factors of UOR electrochemical processes in Ni(OH)_2_ films, offering valuable guidance for the design and enhancement of next-generation electrocatalytic materials.

## Materials and methods

### Chemicals

Chemical reagents including Ni(NO_3_)_2_·6H_2_O, NiCl_2_·6H_2_O, NaOH, KOH, ethanol and isopropanol were procured from Sinopharm Chemical Reagent. All experiments employed deionized and decarbonated water.

### Synthesis of colloid 2D Ni(OH)_2_ nanosheets

The preparation of 2D Ni(OH)_2_ nanosheets was accomplished through a swift ion precipitation method at room temperature. This method involved the utilization of Ni^2+^ and OH^−^ ions, followed by sonication under ambient conditions. Specifically, a mixed solution containing 1.5 mmol of Ni^2+^ metal ions originating from Ni(NO_3_)_2_ was rapidly introduced into a 20 mL aqueous solution of 0.15 M NaOH (20% ethanol) under vigorous stirring.

The resultant precipitate underwent several rounds of centrifugation and washing with deionized (DI) water. Subsequently, the precipitate was subjected to overnight sonication in a mixed solution of water and isopropanol (1 : 1 volume ratio) at room temperature. This procedure yielded a homogeneous and stable suspension of colloidal 2D Ni(OH)_2_ nanosheets (2 mg mL^−1^) exhibiting a positive zeta potential of approximately 27 mV.

### Preparation of Ni(OH)_2_-cF and Ni(OH)_2_-gPF

Calculated quantities of stable colloidal solutions containing 2D Ni(OH)_2_ nanosheets were deposited onto diverse substrates to yield *c*-oriented Ni(OH)_2_ films (Ni(OH)_2_-cF) through solution evaporation-driven self-assembly. The substrates encompassed nickel foils, fluorine-doped tin oxide (FTO), glassy carbon, and monocrystalline silicon sample holders for Powder X-ray Diffraction (XRD).

The thick Ni(OH)_2_-cF films obtained from colloidal solutions of 2D Ni(OH)_2_-cF nanosheets were removed from the substrates and pulverized into powders, denoted as Ni(OH)_2_-gP. Subsequently, Ni(OH)_2_-gP was introduced into a mixed solution of water and isopropanol (1 : 1), followed by sonication to yield a homogeneous dispersion. The resulting dispersion was then deposited onto substrates to generate Ni(OH)_2_-gPF for subsequent applications.

### Characterization

X-ray diffraction patterns were acquired using a Bruker D8-Advance X-ray diffractometer, employing Cu Kα radiation with a wavelength of 1.5405 Å and a scanning rate of 0.02° s^−1^. Monocrystalline silicon sample holders were utilized for sample application, ensuring minimal background diffraction interference. Sample morphologies were scrutinized using a Hitachi S-4800 field emission scanning electron microscope (SEM) and a transmission electron microscope (TEM, FEI Tecnai G2 F20). UV-vis transmittance spectra were captured using a Shanghai Aucy Scientific Instrument UV1902PC UV-vis-NIR spectrophotometer. X-ray photoelectron spectroscopy (XPS) measurements were conducted on a Thermo Fisher-VG Scientific (ESCALAB 250Xi) photoelectron spectrometer. Binding energies were calibrated using adventitious hydrocarbon referencing, with C 1s fixed at 284.60 eV. During the course of UOR, *in situ* Raman spectra (Alpha300R, WETEC, Germany) were employed to monitor the evolution of electrode surface species.

### Electrochemical tests

Electrochemical assessments were executed using an electrochemical workstation (CHI 760E) coupled with a three-electrode cell setup. The working electrode comprised a glassy carbon electrode coated with the electrocatalyst, while a carbon rod served as the counter electrode, the reference electrode adopted was a saturated Hg/HgO electrode. Employing the Nernst equation, potentials were transformed to values relative to the reversible hydrogen electrode (RHE): *E*(RHE) = *E*(Hg/HgO) + 0.098 V + 0.0591 × pH. Linear sweep voltammetry (LSV) traces were systematically recorded using a scan rate of 5 mV s^−1^ in a carefully deoxygenated solution comprising 1 M potassium hydroxide (KOH) blended with 0.33 M urea. All polarization curves were corrected for the *iR* (95%) drop by considering the ohmic resistance of the solution. Operando electrochemical impedance spectroscopy was meticulously conducted employing the Autolab PGSTAT302N instrument from Eco Chemie, Utrecht, Netherlands. This investigation took place within a three-electrode system, with diverse potentials explored across the frequency spectrum ranging from 1 MHz to 0.1 Hz.

For the experimental measurements, a meticulously prepared homogeneous dispersion containing isopropanol, totaling 10 μL in volume, was skillfully deposited onto the surface of a glassy carbon electrode (diameter of 5 mm) bearing an electrocatalyst loading of approximately 0.2 mg cm^−2^. The formation of the Ni(OH)_2_-cF layer on the glassy carbon electrode was achieved *via* a technique driven by solvent evaporation-induced self-assembly. Subsequently, post-evaporation of the solvent, a volume of 2 μL of a 0.5 wt% Nafion solution was deposited onto the electrodes of Ni(OH)_2_-gPF. The determination of the double layer capacitance (Cdl) was executed by measuring cyclic voltammograms (CV) across various scan rates, confined within a non-faradaic potential range spanning 1.13–1.23 V. Notably, the calculated Cdl value corresponds to half of the linear slope in the pertinent region. The overpotential, a parameter of crucial importance, was quantitatively assessed using the ensuing eqn (1):

1 Overpotential = *E*(RHE) − 1.23 V

## Results and discussion

**Fig. 1:**
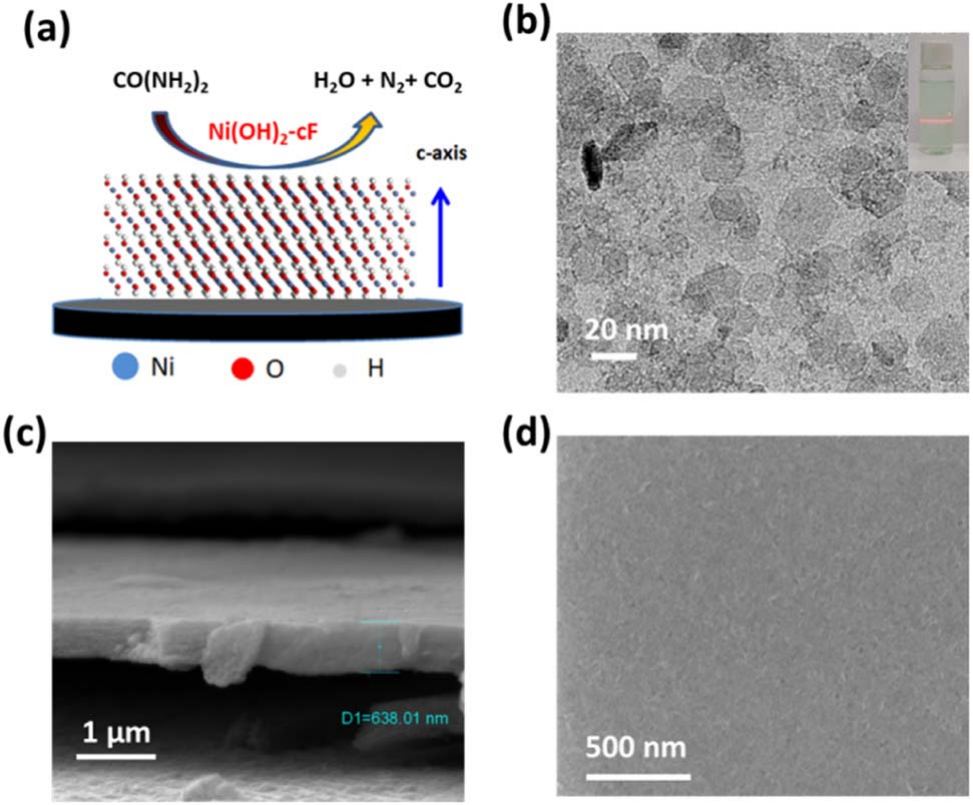
(a) Schematic diagram of self-assembling Ni(OH)_2_ thin film for UOR. (b) TEM image of the as-prepared colloidal 2D Ni(OH)_2_ nanosheets. (c) High-resolution SEM image of the Ni(OH)_2_-cF on the nickel foil. (d) High-resolution top view SEM image of the Ni(OH)_2_-cF.

Fig. 1a presents a conceptual illustration of the self-assembly thin films Ni(OH)_2_-cF used in UOR. The auto-organization procedure of monodispersed and colloidal 2D Ni(OH)_2_ nanosheets, characterized by their atomically-thin features, resulted in the formation of a highly uniform close-packed superlattice exhibiting a *c*-oriented feature. The role of colloidal 2D hydroxide nanosheets as rudimentary building blocks for forming *c*-oriented hydroxide films, as corroborated by numerous scholarly investigations,^30–33^ is achieved through a layer-by-layer self-assembly technique extensively described in the literature. Surface tension, van der Waals interactions, electrostatic and gravitational forces acting upon the colloidal 2D LDH nanosheets influence the self-assembly operation.^33^ A solvent evaporation-induced self-assembly approach enabled us to successfully prepare transparent *c*-oriented films Ni(OH)_2_-cF^27^ on varied substrates, such as nickel foils, fluorine-doped tin oxide (FTO) glass, monocrystalline silicon sample holder and glassy carbon, as evidenced in our investigation. In the presence of Ni(OH)_2_ species as catalysts, urea molecules decompose into H_2_O, N_2_, and CO_2_. The UOR can be represented by CO(NH_2_)_2_ + 6OH^−^ → N_2_ + 5H_2_O + CO_2_ + 6e^−^.^34^

The colloidal 2D Ni(OH)_2_ nanosheets were fabricated using a rapid co-precipitation method with Ni^2+^ and OH^−^ ions, followed by sonication at ambient temperature. The microstructure analysis of the 2D Ni(OH)_2_ nanosheets was conducted by using TEM as illustrated in Fig. 1b. TEM imaging in Fig. 1b demonstrates the successful generation of well-distributed nanosheets with a lateral dimension ranging from 20 to 50 nm. The colloidal solution exhibited remarkable dispersion stability, as evidenced by the observation of the Tyndall effect (inset in Fig. 1b).^35^ This optical phenomenon indicates uniform dispersion of nanosheets in the aqueous medium, which persists even after a storage period of 5 months. The long-term stability of the colloidal solution is particularly significant as it ensures nanosheet integrity and functionality, rendering them suitable for diverse applications.

High-resolution TEM analysis, as demonstrated in Fig. S1,† reveals distinct lattice fringes with a measured lattice spacing of 0.23 nm, corresponding to the (011) lattice planes of the Ni(OH)_2_ crystal structure. SEM image illustrates in Fig. 1c, delineates the measured thickness of Ni(OH)_2_-cF on nickel foil substrates, estimated to be 638 nm, featuring a compactly packed structure. Notably, a remarkably smooth surface morphology without discernible grain boundaries or pores within the film structure is clearly visible (Fig. 1c and d).

**Fig. 2:**
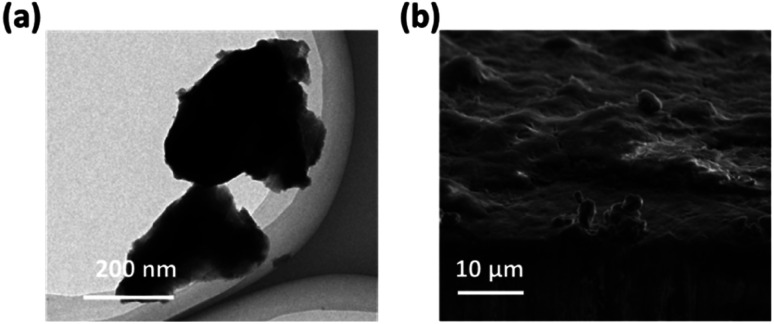
(a) TEM image of the as-prepared Ni(OH)_2_-gP. (b) High-resolution SEM image of the Ni(OH)_2_-cF on the nickel foil.

For comparison, the Ni(OH)_2_-cF samples were pulverized into powders, referred to as Ni(OH)_2_-gP. After ultrasonication, Ni(OH)_2_-gP samples underwent an irreversible transformation from their original colloidal monodisperse 2D Ni(OH)_2_ nanosheet configuration, as evident in Fig. 2a. The high-resolution TEM image of Fig. S2† clearly demonstrates the enhanced crystallinity of Ni(OH)_2_-gP, while Fig. 2a illustrates the presence of predominantly micron-sized large particles. It is evident that the Ni(OH)_2_-gP underwent a dense structural transformation during the self-assembly process. As a result, the resultant films derived from Ni(OH)_2_-gP, referred to as Ni(OH)_2_-gPF, showed significant differences from the characteristics of Ni(OH)_2_-cF. Notably, Ni(OH)_2_-gPF films revealed an irregular morphology and uneven surface, as visually portrayed in Fig. 2b.

**Fig. 3:**
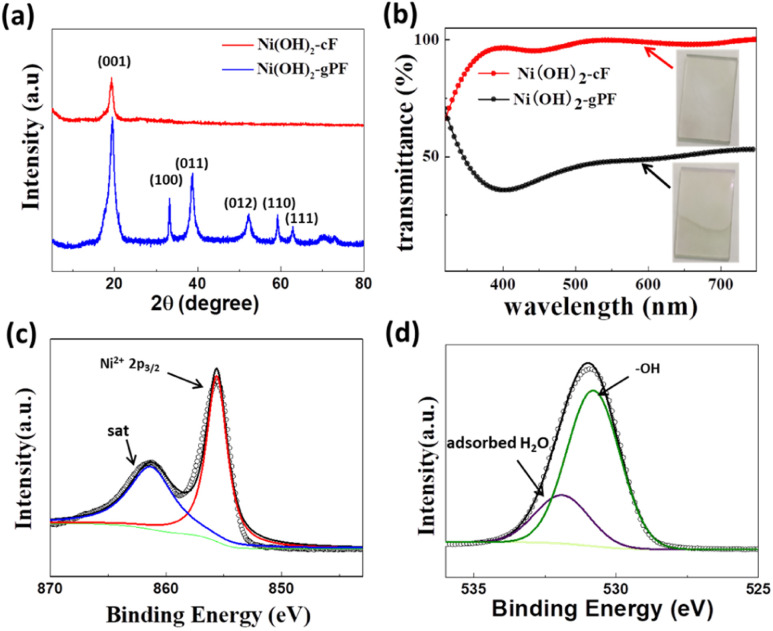
(a) XRD patterns of the as-prepared Ni(OH)_2_-cF and Ni(OH)_2_-gPF on the monocrystalline silicon sample holder. (b) UV-vis transmittance spectra and the photographs (insets) of the Ni(OH)_2_-cF and Ni(OH)_2_-gPF. (c) High-resolution XPS spectra of Ni 2p of the as-prepared Ni(OH)_2_-cF. (d) High-resolution XPS spectra O 1s for the as-prepared Ni(OH)_2_-cF.

The XRD pattern in the Fig. 3a confirms the pronounced preferential orientation of Ni(OH)_2_-cF on the substrates. The XRD pattern (Fig. 3a) of Ni(OH)_2_-cF exhibits exclusively (001) diffraction peaks, indicative of the *c*-oriented attribute. Notably, the absence of any non-basal reflections (*h*, *k* ≠ 0) at high angles denotes an exceptionally well-established *c*-oriented assembly of 2D Ni(OH)_2_ nanosheets.^29,36–38^ In contrast, the XRD pattern of the comparative specimen Ni(OH)_2_-gPF discloses the presence of (012) and (110) peaks, along with other non-*c*-axis oriented peaks (*h*, *k* ≠ 0) at high angles, suggesting the manifestation of diffuse scattering due to irregular stacking.^39^ Additionally, the diffraction peak intensity of Ni(OH)_2_-gPF is significantly elevated relative to that of Ni(OH)_2_-cF, implying good crystallinity in the self-assembled films. These results conclusively ascertain that the crystallites in Ni(OH)_2_-cF are oriented on the substrate with their *c*-axis perpendicular to the substrate surface. The XRD patterns of the transparent *c*-oriented Ni(OH)_2_-cF with different thickness on the FTO substrate (Fig. S3a and S5a†), nickel foil (Fig. S3b and S4†) and glassy carbon (Fig. S5b†) further validate the *c*-oriented hydroxide films fabricated through the colloidal nanocrystal self-assembly approach on these substrates. Furthermore, it is noteworthy that all Ni(OH)_2_-cF samples consistently displayed a lattice parameter of 4.596 nm along [001] direction, closely resembling the value of 4.617 nm reported in the JCPDS database for β-Ni(OH)_2_ (JCPDS file no. 76-1520).

Systematic investigations were conducted into the optical transmittance characteristics of Ni(OH)_2_-cF and Ni(OH)_2_-gPF. Fig. 3b demonstrates that the Ni(OH)_2_-cF film on the FTO substrate exhibits notably enhanced transparency, with an optical transmittance exceeding 90% between 400 nm and 750 nm. This increased transparency can be attributed to the absence or significant decrease in pores amid the orderly self-assembled 2D Ni(OH)_2_ nanosheets, effectively mitigating Mie and Rayleigh scattering effects.^40^ Conversely, the Ni(OH)_2_-gPF film manifests an opaque and rough surface (inset in Fig. 3b), maintaining an optical transmittance approximately 45% across the wavelength range of 400 nm to 750 nm. The augmented transparency of the self-assembled Ni(OH)_2_ film signifies its excellent ordered stacking structure.

X-ray photoelectron spectroscopy (XPS) measurements were performed to acquire insights into the chemical valence states of the Ni(OH)_2_-cF film. The XPS spectrum of Ni 2p_3/2_ (Fig. 3c) can be deconvoluted into two distinct peaks: the peak located at a binding energy of 855.6 eV validates the dominant valence state of Ni as +2, while the peak discerned at a binding energy of 863 eV is attributed to the satellite peak. Moreover, the high-resolution O 1s spectrum (Fig. 3d) of Ni(OH)_2_-cF reveals two characteristic peaks at 530.8 eV and 531.9 eV, attributable to the –OH and the adsorbed H_2_O species, respectively. Consequently, the Ni(OH)_2_-F film demonstrates the same chemical valence states as previously reported for Ni(OH)_2_.^10,13,27,41^

To assess the enhanced UOR electrocatalytic performance of self-assembled *c*-oriented hydroxide films, we conducted a comprehensive investigation of Ni(OH)_2_-cF and Ni(OH)_2_-gPF catalysts on glassy carbon in 1 M KOH electrolytes supplemented with the addition of 0.33 M urea, utilizing a three-electrode configuration at ambient temperature. Importantly, the self-assembled Ni(OH)_2_-cF film demonstrated excellent adhesion to the substrates, whereas Ni(OH)_2_-gPF indicated a propensity to detach from the substrates, mandating the necessary use of Nafion solution on these electrodes. The catalyst loading for all films was uniformly maintained at 0.2 mg cm^−2^.

**Fig. 4:**
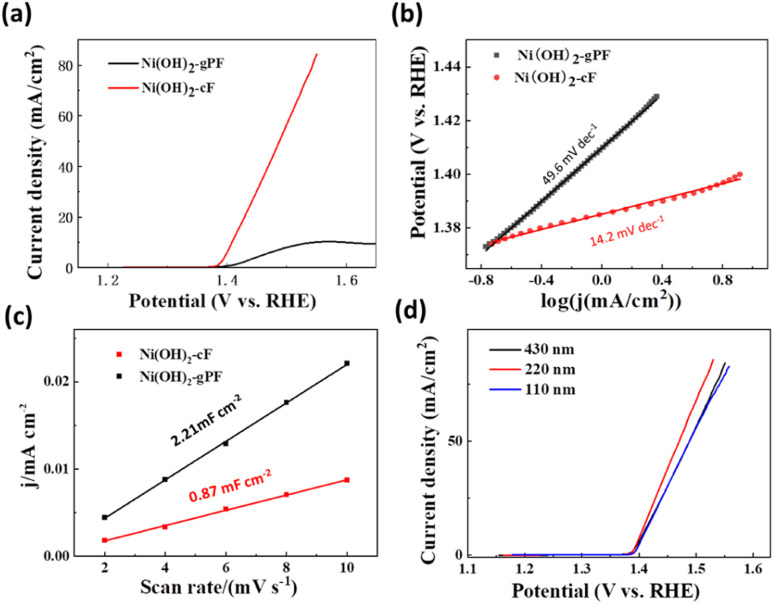
The UOR performance of as-prepared Ni(OH)_2_-cF and Ni(OH)_2_-gPF. (a) *iR*-corrected LSV curves in an N_2_-saturated 1 M KOH electrolyte supplemented with 0.33 M urea at a scan rate of 5 mV s^−1^. (b) Corresponding Tafel slopes. (c) Corresponding electrochemically active surface area. (d) *iR*-corrected LSV curves of Ni(OH)_2_-cF film with different thickness at a scan rate of 5 mV s^−1^.

The Linear Sweep Voltammetry (LSV) measurements depicted in Fig. 4a reveal that the electrocatalytic activity of Ni(OH)_2_-cF towards the UOR markedly transcends that of Ni(OH)_2_-gPF. In the context of overpotentials, Ni(OH)_2_-cF manifested an exceptional electrocatalytic performance, displaying a significantly lower value of 180 mV at a current density of 10 mA cm^−2^, exceeding Ni(OH)_2_-gPF catalysts by 118 mV. Alternatively, within the potential range of 1.4 to 1.55 V for linear sweep voltammetry (LSV) measurements, the oxidation current density of Ni(OH)_2_-cF exhibited a linear increase with applied voltage, while Ni(OH)_2_-gPF demonstrated a conspicuous passivation trend after achieving a current density of 12 mA cm^−2^ at 1.50 V during the UOR. The kinetics of the electrochemical reaction process were scrutinized through Tafel plots (Fig. 4b). Ni(OH)_2_-cF exhibited a significantly smaller Tafel slope (14.2 mV dec^−1^) relative to Ni(OH)_2_-gPF (49.6 mV dec^−1^), implying that the colloid self-assembly *c*-oriented hydroxide film effectively expedited the sluggish kinetics of UOR. The turnover frequency (TOF) represents the figure of merit for intrinsic activity, attributed to all the Ni sites of Ni(OH)_2_-cF. As shown in Fig. S6,† the TOF value (0.135 s^−1^ at 260 mV overpotential) of Ni(OH)_2_-cF is almost 8.4 fold higher than that of Ni(OH)_2_-gPF (0.016 s^−1^), which indicated the strong enhanced UOR activity.

In addition, the electrochemically active surface area (ECSA) of the electrocatalysts was evaluated using electrical-double-layer capacitances (Cdl) calculated from cyclic voltammograms (CVs). As illustrated in Fig. 4c and S7,† in contrast to Ni(OH)_2_-gPF (2.21 mF cm^−2^), it provides additional evidence supporting the smaller Cdl value of 0.87 mF cm^−2^ for Ni(OH)_2_-cF, consistent with the flat surface morphology of Ni(OH)_2_-cF (Fig. 1d). These collective results strongly confirm that the observed high UOR performance of Ni(OH)_2_-cF cannot be singularly ascribed to the active area. The effect of catalyst film thickness on the UOR performance was additionally examined, and we observed that the UOR electrocatalytic activity remained consistently high across sample thicknesses ranging from 96 nm to 430 nm (Fig. 4d). This observation suggests that the electrocatalytic activity of UOR is predominantly concentrated at the surface of the Ni(OH)_2_ material, which aligns with previously documented observations in the literature.^42,43^ The electrocatalytic stabilities of Ni(OH)_2_-cF and Ni(OH)_2_-gPF on the nickel foil substrate were further characterized by the chronoamperometry test. As shown in Fig. S8,† the Ni(OH)_2_-cF could maintain stable current density for 10 h period of UOR testing, and retain 82% current density at a current density of 10 mA cm^−2^ after 10 h period. However, the current densities for Ni(OH)_2_-gPF dropped by 37% respectively over a 10 h period.

**Fig. 5:**
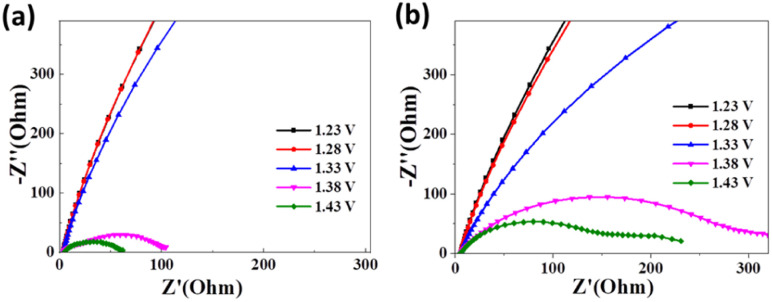
Nyquist plots obtained during *in situ* electrochemical impedance spectroscopy at different voltages for the prepared samples of (a) Ni(OH)_2_-cF and (b) Ni(OH)_2_-gPF.

During *in situ* electrocatalytic operations, Electrochemical Impedance Spectroscopy (EIS) offers a valuable analytical tool for exploring the pivotal parameters directing the UOR electrochemical procedure. Particularly, Nyquist plots within the high-frequency domain deliver crucial intelligence pertaining to solution resistance (*R*_s_) and charge transfer resistance (*R*_ct_), as signified by the radius of the semicircle. As indicated in Fig. 5a for Ni(OH)_2_-cF, the semicircle's diameter diminishes with the application of a bias voltage, achieving a minimum value of 101 Ohm at 1.38 V and 61 Ohm at 1.43 V, both significantly lower than Ni(OH)_2_-gPF (Fig. 5b). These EIS results are consistent with the observations of reduced overpotential and minimal Tafel slope, further substantiating the catalytic superiority of the self-assembled *c*-oriented Ni(OH)_2_ film. Additionally, a noticeable second semicircle appears at 1.53 V for Ni(OH)_2_-gPF (Fig. 5b), typically due to the inception of strongly adsorbed intermediates.^44–47^ This observation may explain the discernable passivation effect noticed in Ni(OH)_2_-gPF,^45^ evidencing that self-assembled *c*-oriented Ni(OH)_2_ films proficiently mitigate the genesis of such intermediates and the passivation trend.

**Fig. 6:**
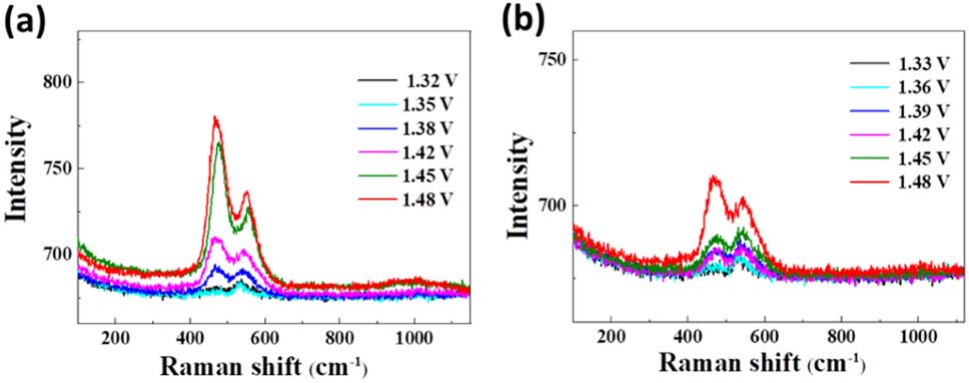
*In situ* Raman spectroscopy at different voltages for the prepared samples of (a) Ni(OH)_2_-cF and (b) Ni(OH)_2_-gPF.

In order to fathom the advantageous electrocatalytic activity of Ni(OH)_2_-cF in urea oxidation, *in situ* Raman spectroscopy (as shown in Fig. 6) was employed as a valuable tool for further probing the key factors influencing the electrochemical UOR process. In every occurrence, the E_g_–Ni–O bending vibration (*ν̃* bend Ni^3+^) within the range of 475–494 cm^−1^ and the A_1g_–Ni–O stretching vibration (*ν̃* str Ni^3+^) within the range of 558–562 cm^−1^ are clearly observed, thus confirming the presence of NiOOH,^48^ in concurrence with the electrochemical findings. As portrayed in Fig. 6, the Ni(OH)_2_-cF sample exhibits the NiOOH Raman signal at 1.42 V, whereas the Ni(OH)_2_-gPF sample displays the NiOOH Raman signal at 1.48 V. This observation implies that Ni(OH)_2_-cF is relatively more susceptible to transform into NiOOH, thereby generating an increased number of catalytic active sites. It's noteworthy that the oxidation potential of Ni(OH)_2_ in this environment is larger than in the three-electrode solution, given the electrolyte in the Raman *in situ* environment is relatively scarce, culminating in a delay in the oxidation potential.

**Fig. 7:**
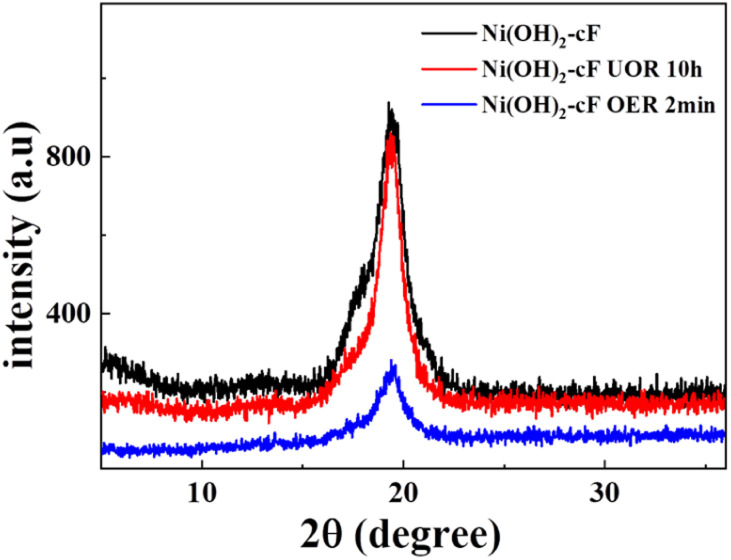
XRD patterns of the as-prepared Ni(OH)_2_-cF on nickel foil before and after various electrochemical reactions.

To garner further insights into the electrocatalytic mechanism of urea, a thorough examination of XRD spectra of Ni(OH)_2_ films after UOR and OER was undertaken. The XRD data collated from samples at diverse time intervals provided valuable insights. As delineated in Fig. 7, after subjecting the system to 2 hours of urea electrooxidation, the lattice structure of Ni(OH)_2_ manifested minimal modifications.

Conversely, a significant change in crystallographic integrity was discerned after a mere 2 minutes of electrocatalytic oxygen evolution, as validated by a diminution in the intensity of the (001) peak and an amplification in peak broadening, signaling structural disruption in the aftermath of the OER catalytic reaction process.^49^ This comparison further confirms that the UOR activity of Ni(OH)_2_ films primarily converges at the surface, and due to the sustained reductive influence of urea on trivalent nickel, the internal structure of the film remains unchanged during the catalytic operation.

## Conclusions

In summary, this study represents a comprehensive exploration of the electrocatalytic activity of self-assembled *c*-oriented Ni(OH)_2_-cF films in the context of UOR, with Ni(OH)_2_-gPF serving as a comparative reference. The comprehensive analysis showed that the Ni(OH)_2_-cF samples demonstrated a pronounced electrocatalytic efficiency, especially when compared to Ni(OH)_2_-gPF, characterized by a significant potential reduction of 180 mV at a current density of 10 mA cm^−2^, exceeding Ni(OH)_2_-gPF by a notable margin of 118 mV, and characterized by smaller Tafel slopes. Additionally, through the implementation of *in situ* EIS analysis and Raman spectroscopy, this investigation has revealed the remarkable propensity of Ni(OH)_2_-cF to transform into NiOOH under *in situ* conditions, providing strong evidence for its outstanding catalytic performance. Overall, this research has provided valuable insights into the underlying mechanisms of UOR electrochemical processes with Ni(OH)_2_, firmly establishing the electrocatalytic advantages of self-assembled *c*-oriented Ni(OH)_2_ films.

## Conflicts of interest

There are no conflicts to declare.

## Supplementary Material

RA-013-D3RA05538H-s001
