# Supplementary material for: Self-assembled c-oriented Ni(OH)2 films for enhanced electrocatalytic activity towards the urea oxidation reaction
Source: RSC Adv. 2023 Oct 10;13(42):29625–31. doi: 10.1039/d3ra05538h (PMC10562896; doi:10.1039/d3ra05538h)
Supplement: RA-013-D3RA05538H-s001 [file RA-013-D3RA05538H-s001.pdf]

Electronic Supplementary Material (ESI) for RSC Advances.
This journal is © The Royal Society of Chemistry 2023

Electronic Supplementary Information

Self-Assembled *c*-Oriented $Ni(OH)_2$ Films for Enhanced Electrocatalytic Activity towards Urea Oxidation Reaction

Xinwei Dong,* Yansheng Liu, Jin Zhou, Guoxiao Xu, Fei Guo, Xiaobo Jia, and Zhiqiang Yu

School of Electronic Engineering, Liuzhou Key Laboratory of New Energy Vehicle Power Lithium Battery, Guangxi University of Science and Technology, Liuzhou 545006, Guangxi, China.

.

Figures S1-S8：

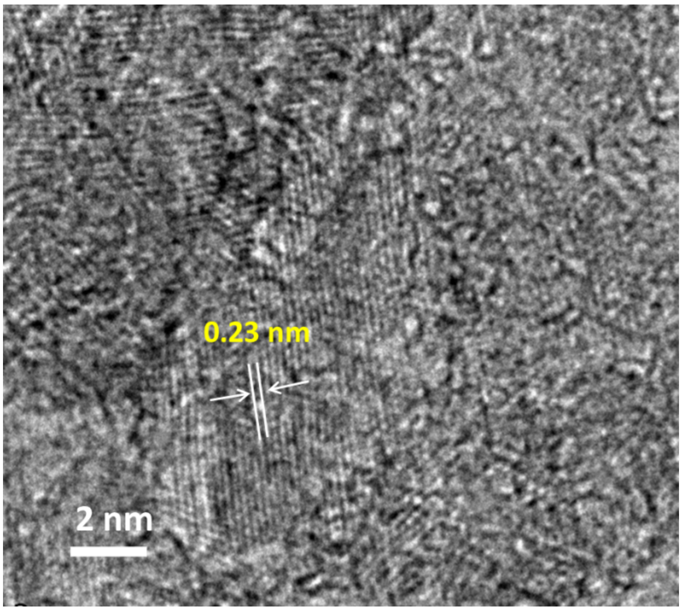

Fig.S1 High-resolution TEM image of as-prepared $Ni(OH)_2$ nanosheets.

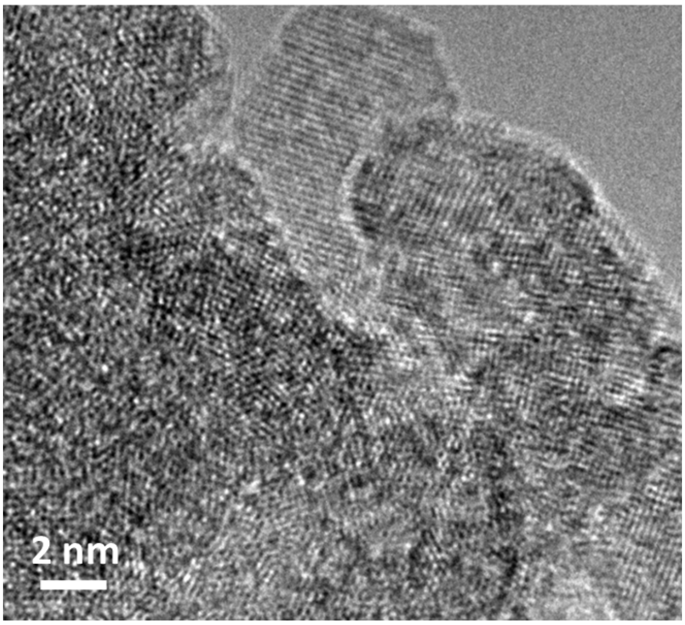

Fig.S2 High-resolution TEM image of as-prepared $Ni(OH)_2$-gP.

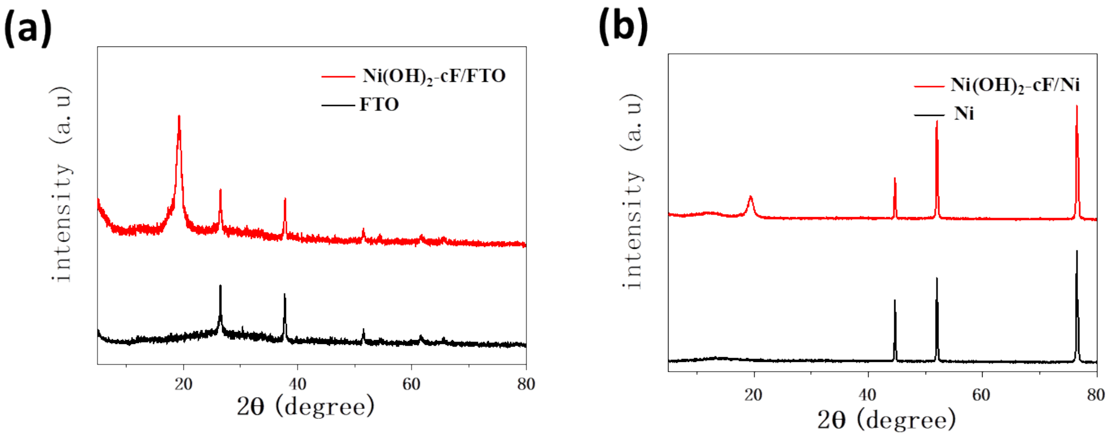

Fig.S3 XRD of the as-prepared $Ni(OH)_2$-cF on the (a) FTO substrate and (b) nickel foil.

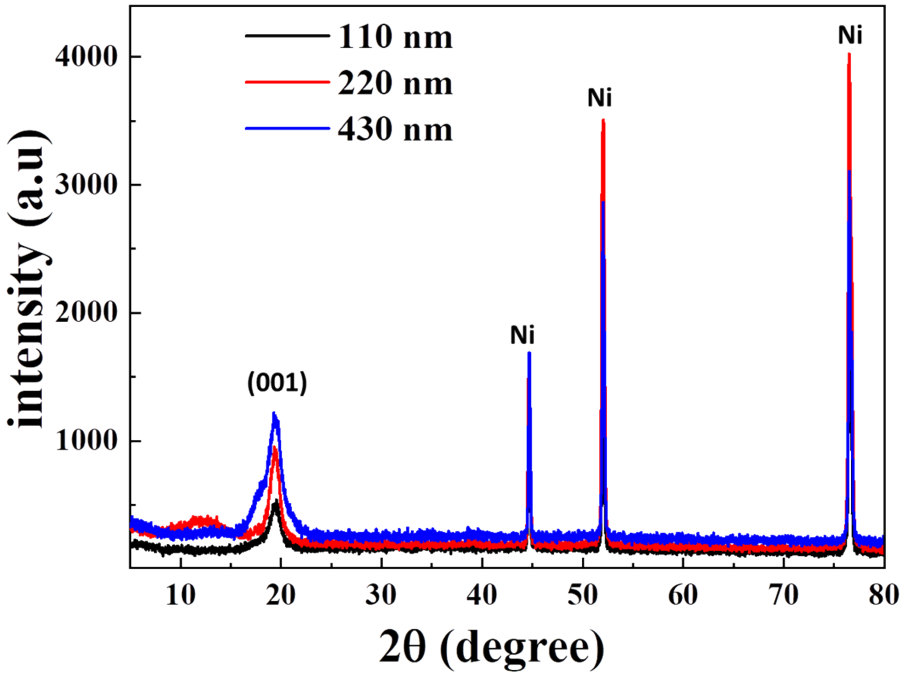

Fig.S4 XRD of the as-prepared $Ni(OH)_2$-cF with different thicknesses on the nickel foil.

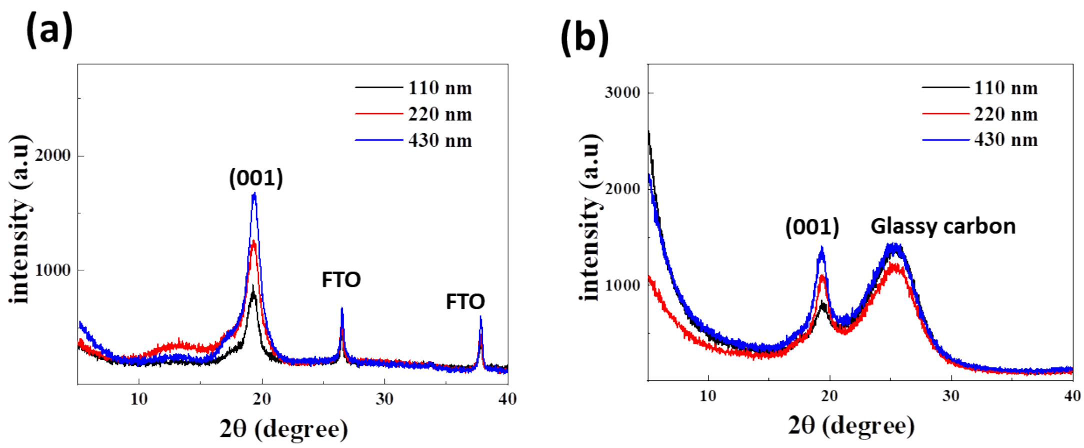

Fig.S5 XRD of the as-prepared $Ni(OH)_2$-cF with different thicknesses on the (a) FTO substrate and (b) glassy carbon.

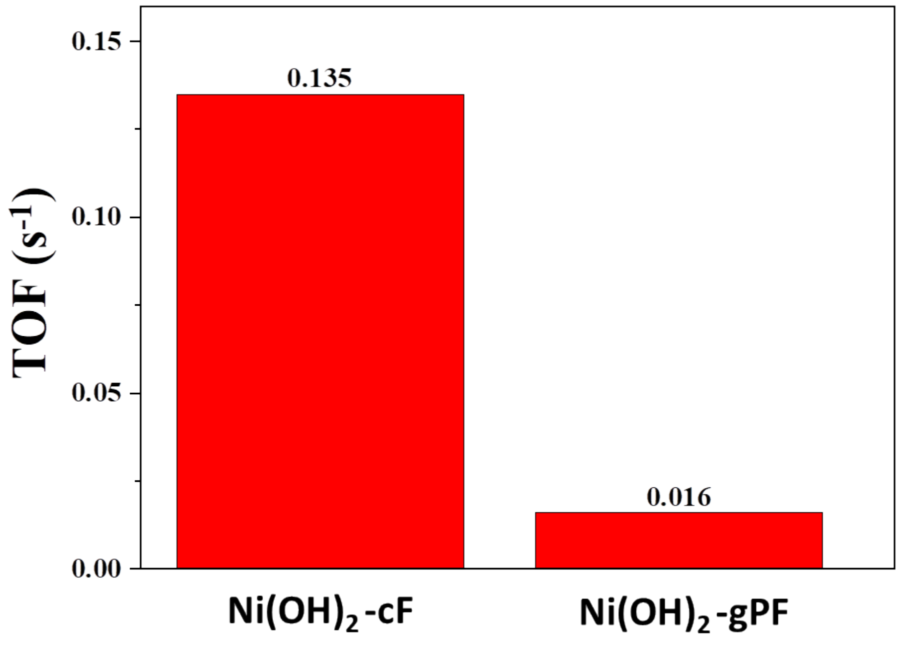

Fig.S6 The TOF values of $Ni(OH)_2$-cF and $Ni(OH)_2$-gPF at η=260 mV.

The TOF values (*Angewandte Chemie, 2020, 132.21: 8149-8154*) were calculated from the following equation:

$$TOF = J * A/4 * F * m$$

where J (A cm^{-2}) is the current density at overpotential of 260 mV, A is the geometric surface area of the electrode, F is the faraday constant (96485 C mol^{-1}), m is the mole number of Ni atoms in the catalyst.

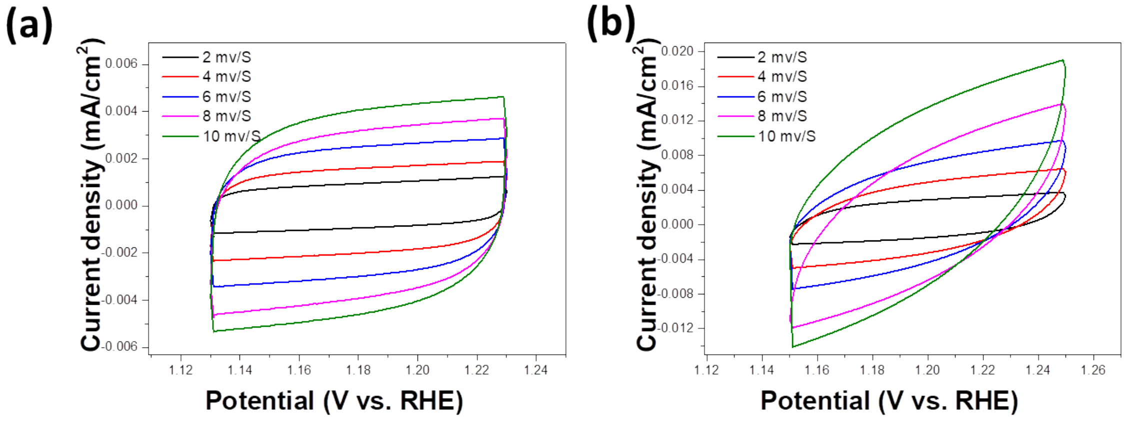

Fig.S7 Typical CV curves at different scan rates of (a) $Ni(OH)_2$-cF and (b) $Ni(OH)_2$-gPF.

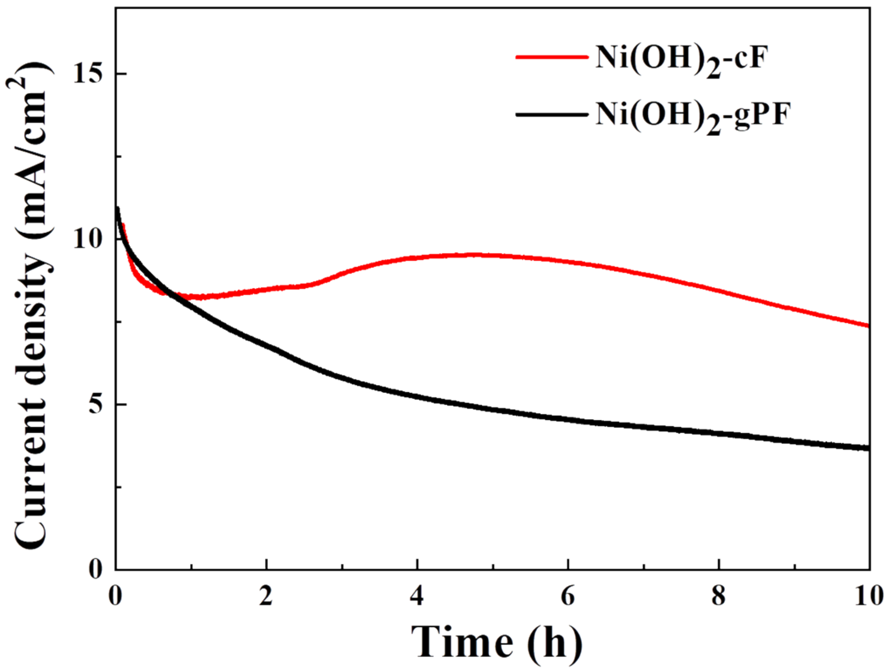

Fig.S8 Chronoamperometry curves at 10 mA/cm^2 of $Ni(OH)_2$-cF and $Ni(OH)_2$-gPF on the nickel foils.
